# Additional morphological characters of the nematode *Capillostrongyloides fritschi* (Travassos, 1914) Moravec, 1982 (Nematoda: Capillaridae) from *Bagrus* spp. (Bagridae) inhabiting Damietta Branch of the River Nile, Egypt with a special reference to evaginated cirrus

**DOI:** 10.1007/s11230-023-10094-4

**Published:** 2023-05-01

**Authors:** Hend E. Allam, Mohamed I. Mashaly, Mohamed M. EL-Naggar

**Affiliations:** Department of Zoology, Faculty of Science, Mansoura University, Mansoura, Egypt

## Abstract

The catfish *Bagrus bajad* (Forsskål) and *B. docmak* (Forsskål) (Siluriformes: Bagridae) are well distributed in the River Nile and have an economic value with good marketing and use in aquaculture. Collected specimens of *Capillostrongyloides fritschi* (Travassos 1914) were redescribed using a phase-contrast microscope and scanning electron microscope. The most remarkable finding was the unique structure of the evaginated cirrus, being composed of a proximal long tube, a middle spherical bulb packed with mature spermatozoa, and a distal funnel-shaped structure. All regions of the cirrus are provided with longitudinal and transverse muscles and covered with transverse cuticular folds. The mechanism of cirrus emergence was discussed based on available musculature data. Other important findings are the short stylet of the mouth, the two lateral oral lobes, 6-10 buried cephalic papillae and the bacillary bands that lack elevations. Stichosome is formed of 35-44 stichocytes that were differentiated into black and white forms, each containing dense granules and translucent vacuoles. The intestine showed a convoluted part at its anterior region. The vulvar lips are slightly elevated, many mature eggs are cleaved, and their shells are 3-layered. The anus was found in a groove bounded by two unequal lobes.

## Introduction

The River Nile freshwater fish are important animal protein sources (Samy-Kamal, 2015). The catfish *Bagrus bajad* (Forsskål) and *B. docmak* (Forsskål) (Siluriformes: Bagridae) are well distributed in the River Nile and have an economic value with good marketing and use in aquaculture (Mashaly et al., 2019). In a recent ecological study of helminth parasites of *Bagrus* spp. from the Damietta Branch of the River Nile, Allam et al. (2023) recorded a nematode species, *Capillostrongyloides fritschi* (Travassos, 1914) Moravec, 1982 (Nematoda: Capillaridae) from the stomach and intestine. Moravec (2001) considered the genus *Capillostrongyloides* as one of the allegedly valid genera in the family Capillariidae. The genus currently includes 10 valid species, namely, *C. fritschi*, *C. sentinosa* Travassos, 1927, *C*. *tandani* Johnston & Mawson, 1940; *C. physiculi* Johnston & Mawson, 1945, C. *tasmanica* Johnston & Mawson, 1945, C. *ancistri* Moravec, Gelnar & Řehulka, 1987, C. *norvegica* Moravec & Karlsbakk, 2000, *C*. *arapaimae* Santos, Moravec & Venturieri, 2008, *C*. *congiopodi* Cantatore, Rossin, Lanfranchi & Timi, 2009, and *C. morae* González-Solís et al., 2014. All species were recorded from the digestive tract of fishes from different regions all over the world: Egypt, Norway, Tasman Sea, Pacific Ocean, Brazil, South Australia, Mexico, Brazilian Amazon (Moravec, 2001; Santos et al., 2008). Species of the genus *Capillostrongyloides* are characterized by a stichosome with a single row of stichocytes, the presence of a well-developed bursa supported by two short and broad lateral lobes, each of them bearing a papilla, and a non-spiny spicular sheath (Moravec, 2001). In Egypt, *C. fritschi* was described by Tadros & Mahmoud (1968) as *Capillaria yamagutii* from the stomach of *B. bajad,* and *B. docmak.* Imam et al. (1991) reported the same species from the same host and locality while Moravec (1974, 2001) revised the same nematode species and considered it a synonym of *C. fritschi*.

Previous descriptions of *C. fritschi* are incomplete since they have been concentrated on the morphometrics and little is known of the structural details of the cephalic region, digestive system, and reproductive system of both females and males, particularly the cirrus (spicular sheath) and vulva. The present study aims to redescribe the capillariide *C*. *fritschi* using the phase-contrast microscope and scanning electron microscopy. Special attention has been given to the cirrus, in invaginated and evaginated positions since they are highly used for the differentiation of the congeneric species.

## Materials and Methods

A total of 141 individuals of the freshwater catfishes *Bagrus bajad* and *Bagrus docmak* were collected from the Damietta branch of the River Nile at Mansoura city from March 2020 to February 2021. Living captured fish were transported in an aerated container, with fresh water, to the parasitology laboratory at Mansoura University and kept alive in an aquarium with aerated water. The parasites were collected and examined following the method of Allam et al. (2023) while their identification and measurements were done according to Moravec (2001). All measurements and scale bars were calculated on 20 living adults using OMAX TopView 3.7 program. For scanning electron microscopy (SEM), 10 living adults were preserved, postfixed, and dehydrated according to El-Naggar et al., (2016). Critical point drying was performed using EMS Q 850 critical point dryer with carbon dioxide as the transition fluid. The specimens were then coated with gold using SPI Sputter Coater and examined with a JEOL JSM 6510 lv electron microscope at the Electron Microscope Unit (EMU) of Mansoura University.

## Results

**Species:** *Capillostrongyloides fritschi* (Travassos, 1914**)** Moravec, 1982

Syn.: *Trichosoma papillosum* Fritsch, 1886, nec Wedl, 1856: *Capillaria fritschi* Travassos, 1914: *C*. *yamagutii* Tadros & Mahmoud, 1968.

**Hosts:** *Bagrus bajad* and *B. docmak* (Bagridae)**.**

**Habitat:** Stomach and intestine

**Locality:** Damietta branch of the River Nile at Mansoura City in Dakahlia Governorate (coordinates: (31°02'47.2"N, 31°21'12.2"E), Egypt.

**Date:** March 2020 -February 2021F

**Prevalence:** 12.95%.

### Description

**Fig. 1:**
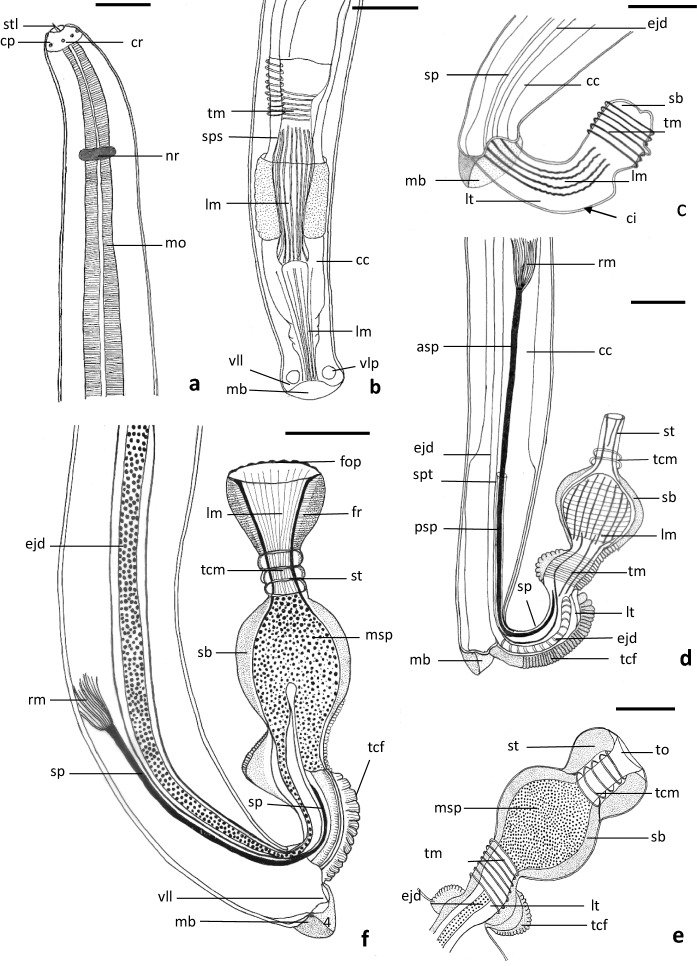
Schematic drawing showing morphological details of male *Capillostrongyloides fritschi* (Travassos, 1914) Moravec, 1982 **a.** Anterior region. **b.** Posterior region (ventral view). **c.** First pattern of everted cirrus. **d.** Second-everted cirrus pattern. **e.** Third-everted cirrus pattern. **f.** Fourth-everted cirrus pattern. Scale bars: **a** and **b** = 30 µm; **c** and **e** = 10 µm; **d** and **f** = 20µm. **Abbreviations: asp,** anterior region of spicule; **cc**, cloaca; **ci**, cirrus; **cp**, cephalic papillae; **cr**, cephalic region; **ejd**, ejaculatory duct; **fop**, opening of funnel-shaped region; **fr**, funnel-shaped terminal region; lm, longitudinal muscles; **lt**, long tube; **mb**, membranous bursa; **mo**, muscular oesophagus; **msp**, mature spermatozoa; **nr**, nerve ring; **psp**, posterior region of spicule; rm, retracted muscles; **sb**, spherical bulb; **sp**, spicule; **sps**, spicular sheath; **spt**, spicular tube; **st**, short tube; stl, finger-like stylet**; tcf**, transverse cuticular folds; **tcm**, transverse circumferential sphincter muscle; **tm**, transverse muscle; **to**, terminal opening; **vll**, ventrolateral lobe; **vlp**, ventrolateral papilla.

**Fig. 2:**
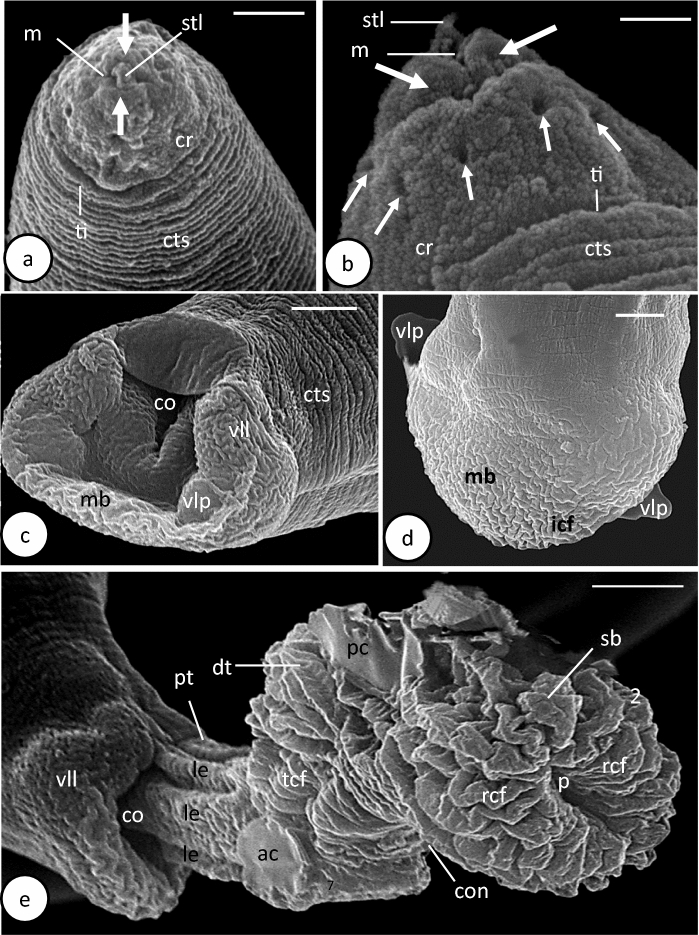
Scanning electron microscope photomicrographs of male *Capillostrongyloides fritschi* (Travassos, 1914) Moravec, 1982 **a.** Cephalic region (frontal view). **b.** Cephalic region (lateral view). **c.** Caudal end (ventral view). **d.** Caudal end (Dorsal view). **e.** Caudal end (evaginated spicular sheath). Scale bar: **a** and **d** = 2 µm; **b =** 1 µm;** c** = 3 µm; **e** = 5µm. Abbreviations: **ac**, anterior concavity; **co**, cloacal opening; **con**, constriction between long cylindrical tube and spherical bulb; **cr**, cephalic region; **cts**, cuticular transverse striations; **dt**, distal wider region; **icf**, irregular cuticular folds; **le**, longitudinal elevations; **m**, mouth opening; **mb**, membranous bursa; **p**, pore; **pc**, posterior concavity; **pt**, proximal region of long cirrus tube; **rcf**, radial cuticular folds; **sb**, spherical bulb; **stl**, finger-like stylet; **tcf,** transverse cuticular folds; **ti**, transverse incision; **vll**, ventrolateral lobe; **vlp**, ventrolateral papilla; **small white arrow**, buried cephalic papilla; **large white arrow**, oral lobe.

**Fig. 3:**
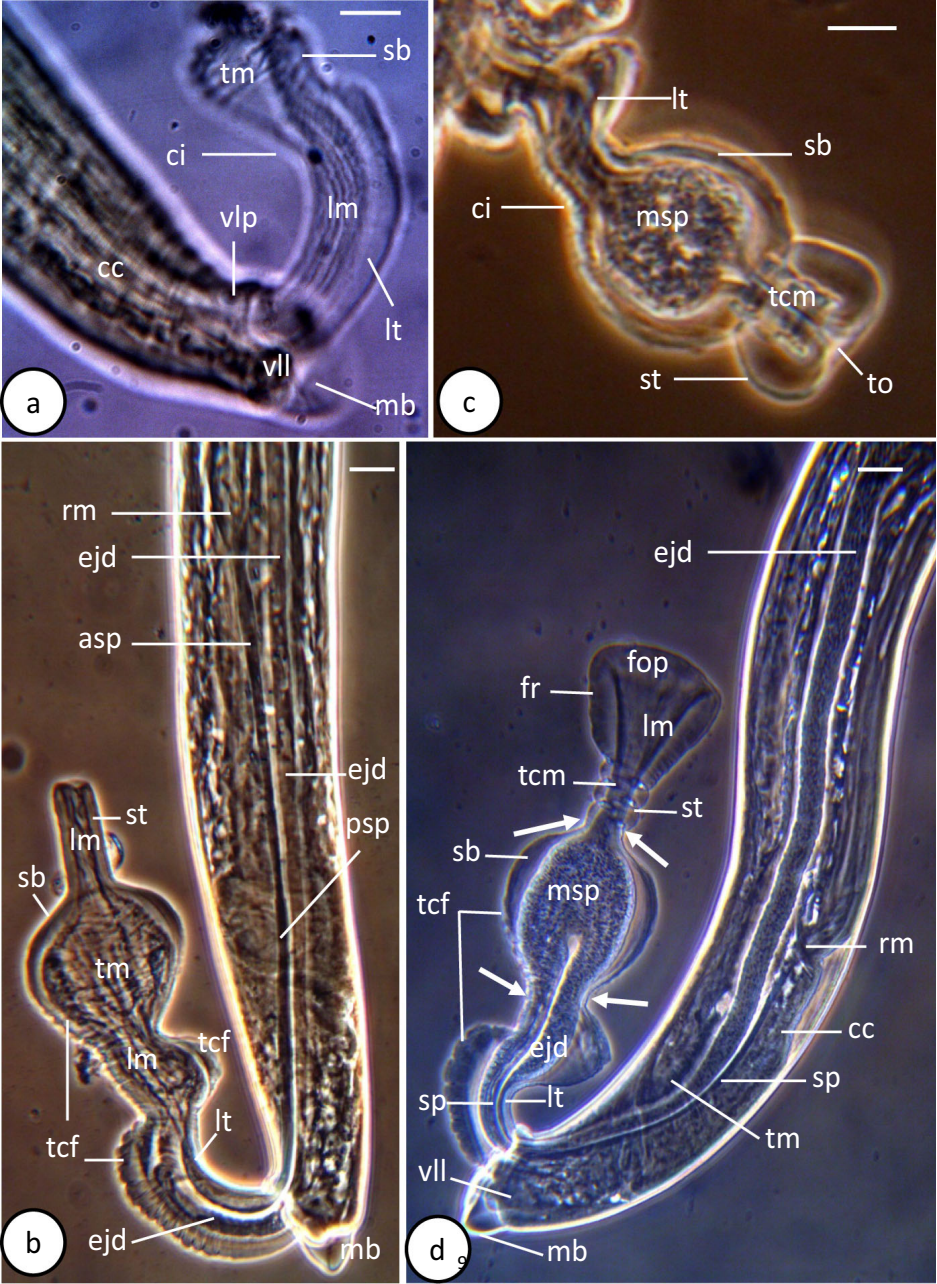
Phase-contrast microscope photographs of male *Capillostrongyloides fritschi* (Travassos, 1914) Moravec, 1982 **a.** First-everted cirrus pattern. **b.** Second-everted cirrus pattern. **c.** Third-everted cirrus pattern. **d.** Fourth-everted cirrus pattern. Scale bars: **a-d** =10µm. Abbreviations: **asp**, Anterior region of spicule; **cc**, cloaca; **ci**, cirrus; **ejd**, ejaculatory duct; **fop**, funnel-shaped opening; **fr**, funnel-shaped terminal region; **lm**, longitudinal muscle; **lt**, long tube; **mb**, membranous bursa; **msp**, mature spermatozoa; **psp**, posterior region of spicule; **rm**, retracted muscle; **sb**, spherical bulb; **st**, short tube; **tcf**, transverse cuticular folds; **tcm**, transverse circumferential sphincter muscles; **tm**, transverse muscles; **to**, terminal opening; **vll**, ventrolateral lobe; **arrow**, constriction.

**Male (**Figs. 1,2,3**)**

Length 3564±608 µm and maximum width 37±20 µm. Cephalic region cone-shaped and separated from body prober by transverse incision (Figs. 1a, 2a). Mouth slit-like structure surrounded by 2 lateral lobes and possessing short finger-like stylet, 1±0.4 µm long (Figs. 1a,2a, b). Amphids not visible. Six to 10 buried small cephalic papillae visible around mouth (Fig. 2 a, b). Head region with regular transverse cuticular striations (Fig. 2 a, b). Total length of oesophagus 1810±129 µm (50.7% of total body length). Muscular oesophagus 202±16 µm long; glandular oesophagus(stichosome) 1608±129 µm long and 31±5 µm width. Stichosome consisting of 35-37 stichocytes, each with large nucleus and dense bodies and superficially subdivided into 4-6 transverse annuli. Two large pseudocoelomic gland cells at oesophagus-intestine junction. Nerve ring 55±10 µm from anterior extremity (1.5% of body length) (Fig. 1a). Lateral bacillary bands extend along whole-body length, number of bacillary cells at middle region greater than anterior and posterior regions. Testis begins at level of oesophagus-intestine junction. Spicule relatively long, 195±45 µm long, with thin tube around its posterior 2 thirds and retractor muscles at its proximal extremity (Figs. 1d, f, 3b, d). Ejaculatory duct 297±21 µm long, passing through cloaca along its length to posterior extremity of body (Figs. 1d, f,2 b, d). Posterior body region provided with well-developed transverse muscle bands, particularly at middle region of spicule (Fig. 3d). Caudal end rounded and provided with well-developed membranous bursa supported by 2 wide, ventrolateral lobes (Figs. 1b, 2c, d,3a). One relatively large papilla present at base of each lobe (Fig. 2 c, d). Surface of ventrolateral lobes and membranous bursa covered with corrugated cuticle (Fig. 2c, d). In living male specimens, evaginated (everted) spicular sheath (cirrus detected in 4 sequent patterns of emergence (Figs. 1c-f, 2e, 3a-d). In first pattern, cirrus emerging as long, cylindrical tube supported by longitudinal muscle and ending with small bulb provided with transverse circumferential muscles (Figs. 1c, 3a). In second pattern, cirrus consisting of long cylindrical tube (proximal region) containing terminal part of spicule and ejaculatory duct, large spherical bulb (middle region), and elongated narrow, terminal tube (distal region) (Figs. 1d, 3b). Surface of both tube and bulb forming cuticular transverse folds while surface of terminal tube smooth (Figs. 1d, 3b). Transverse circumferential and longitudinal muscles present in both long tube and spherical bulb (Figs. 1d, 3b). Terminal tube with terminal opening and supported by longitudinal muscles (Fig. 3b). Third pattern showing more conspicuous transverse circumferential muscles around internal region of terminal tube and dense masses of probably mature spermatozoa filling bulb cavity (Figs. 1e, 3c). In fourth pattern, fully everted cirrus clearly seen at 223±31 long (Figs. 1f, 2e, 3d). Terminal tube expanding into funnel-shaped region (distal region) supported by longitudinal and fine transverse muscle fibres (Figs. 1f, 3d). Constrictions found between long tube and spherical bulb and between spherical bulb and short tube (Figs. 1f, 2e, 3d). Mature spherical-shaped spermatozoa filling bulb cavity (Figs. 1f, 3d).

SEM showed that the everted cirrus is not completely evaginated and only the long cylindrical tube and spherical bulb are seen emerging from the cloacal opening (Fig. 2e). None of the short tube and funnel-like regions were seen in this specimen. The basal part of the tube is relatively narrow and formed of parallel longitudinal elevations that probably represent underneath longitudinal muscles (Fig. 2e). The distal part of the tube, which is wider than the basal one, is covered by transverse cuticular folds and shows two circular, smooth, concavity-like structures (anterior and posterior) (Fig. 2e). The bulb region, which is wider than the cylindrical tube, is covered with considerably large, radially orientated cuticular folds and terminates with a single median pore (Fig. 2e).

**Fig. 4:**
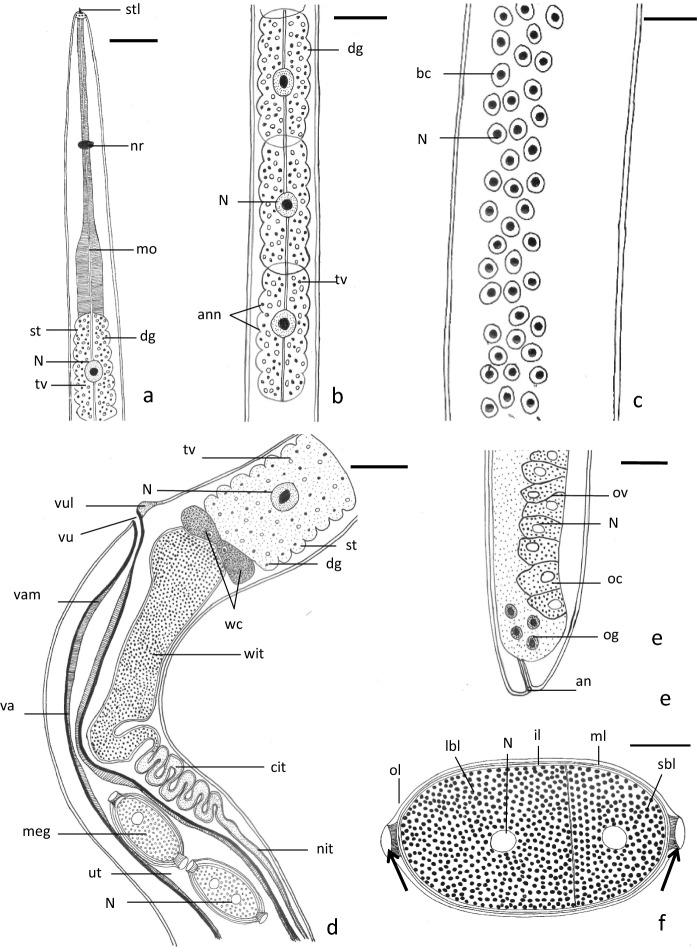
Schematic drawing of female *Capillostrongyloides fritschi* (Travassos, 1914) Moravec, 1982 **a**. Anterior body region. **b.** Stichosome. **c.** Bacillary band. **d.** Anterior region of intestine and distal region of reproductive system. **e.** Caudal body region. **f.** Mature egg. Scale bars: **a**, **d** and **e** = 30µm. **b** and **c** = 20 µm, **f** = 10 µm. Abbreviations**: an**, anus; **ann**, annuli; **bc**, bacillary cell; **cit**, convoluted intestine; **dg**, dense granule; **il**, inner layer; **lbl**, large blastomere; **mo**, muscular oesophagus; **meg**, mature egg; **ml**, middle layer; **N**, nucleus; **nit**, narrow intestine; **nr**, nerve ring; **oc**, oocyte; **og**, oogonium; **ol**, outer thick layer; **ov**, ovary; **sbl**, small blastomere; **st**, stichosome; **stl**, finger-like stylet; **tv**, translucent vesicles; **ut**, uterus; **va**, vagina; **vam**, vaginal muscles; **vu**, vulva; **vul**, vulvar lip; **wc**, wing-like cells; **wit**, widened region of intestine; **arrow**, opercular plug.

**Fig. 5:**
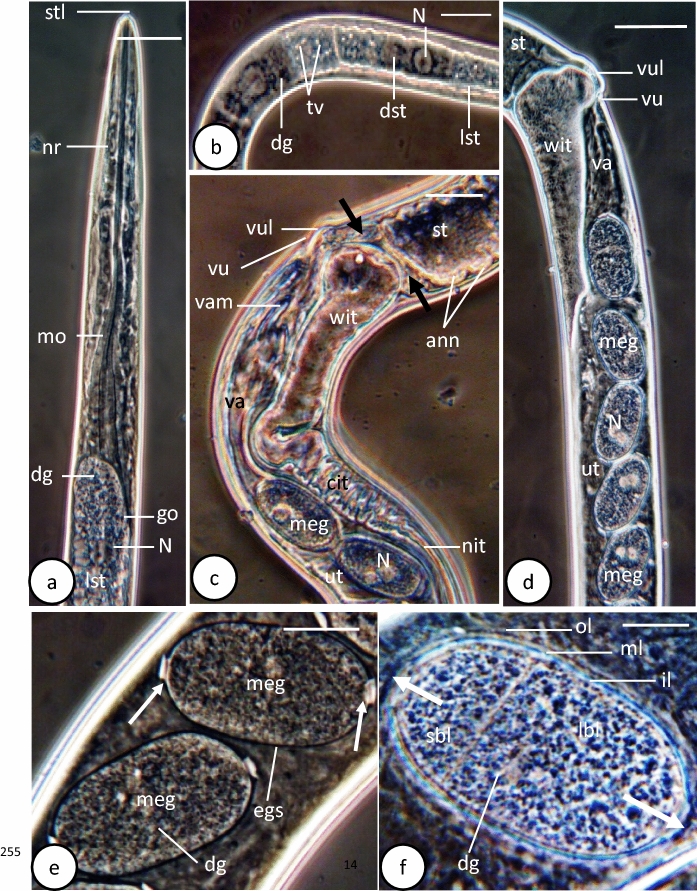
Phase-contrast microscope photographs of female *Capillostrongyloides fritschi* (Travassos, 1914) Moravec, 1982 **a.** Anterior body region. **b**. Stichosome. **c.** Anterior region of intestine and distal region of reproductive system. **d**. Distal portion of uterus with single row of mature cleaved eggs. **e.** Mature eggs with polar plugs (small white arrows). **f.** Mature egg with large (lbl) and small (sbl) blastomeres. Scale bars: **a** and **c** = 30µm, **b** and** d** = 50 µm, **e** = 20µm,** f** =10 µm. Abbreviations: **ann**, annuli; **cit**, convoluted intestine; **dg**, dense granule; **dst**, dark stichocyte; **egs**, eggshell; **go**, glandular oesophagus; **il**, inner thin layer; **lbl**, large blastomere; **lst**, light stichocyte; **meg**, mature egg; **ml**, middle hyaline layer; **mo**, muscular oesophagus; **N**, nucleus; **nit**, narrow intestine; **nr**, nerve ring; **ol**, outer thick layer; **sbl**, small blastomere; **st**, stichosome; **stl,** finger-like stylet; **tv**, translucent vesicles; **ut**, uterus; **va**, vagina; **vam**, vaginal muscles; **vu**, vulva; **vul**, vulvar lip; **wit**, widened region of intestine; **white arrow**, two polar plugs; **black arrows**, wing-like cells.

**Fig. 6:**
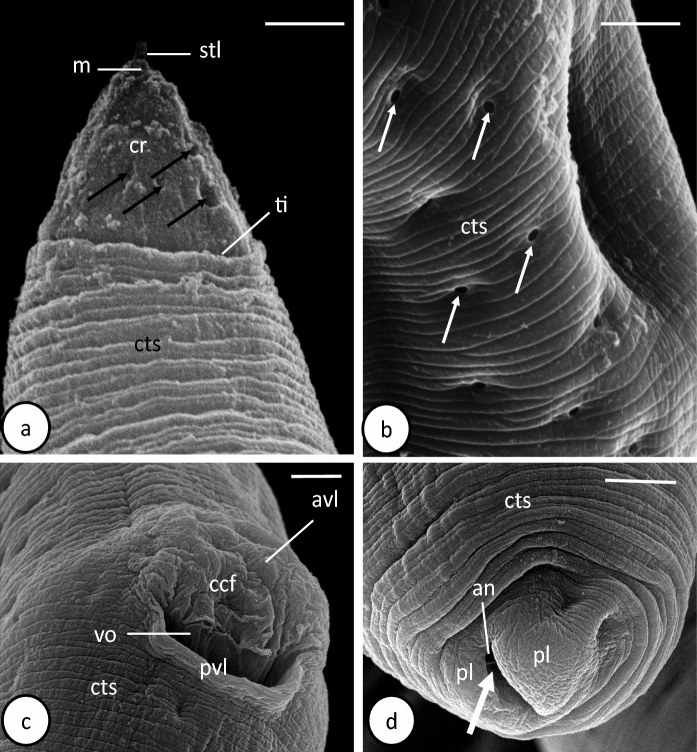
Scanning electron microscope photomicrographs of female *Capillostrongyloides fritschi* (Travassos, 1914) Moravec, 1982 **a.** Cephalic region (ventral view).. **b.** Bacillary bands (lateral view). **c.** Vulva (ventral view). **d.** Caudal body region (ventrolateral view). Scale bars: **a = 2 µm, b,c and d = 5 µm**. Abbreviations: **an**, anus; **avl**, anterior vulvar lip; **ccf**, corrugated cuticular folds; **cr**, cephalic region; **cts**, cuticular transverse striations; **m**, mouth; **pl**, posterior lobe; **pvl**, posterior vulvar lip; **stl**, finger-like stylet; **ti**, transverse incision; **vo**, vulvar opening; **small white arrow**, circular openings of underneath bacillary cells; **small black arrow**, buried cephalic papillae; **large white arrow**, long groove.

**Female (Figs.** **4**, **5**, **6****)**

Total length 5440±1770 µm, anterior width 38±13 µm and maximum width 55±23 µm. Mouth small, slit-like, with very short stylet (Figs. 4a, 5a, 6a). Oral lobes invisible (Fig. 6a). Length of small stylet (2±0.6) µm. Entire length of oesophagus 2147±1320 µm (39.4% of total body length), short muscular part 185±82 µm and posterior long glandular part 1962±1320 µm. Muscular oesophagus narrow at its anterior half and wide at its posterior half (Figs. 4a, 5a). Stichosome formed of single longitudinal row of 40-44 large stichocytes, subdivided superficially into 6-8 transverse annuli (Figs. 4b, 5b). Two types of stichocytes, 4 light-coloured cells, 53±7 µm long, found anteriorly, followed by 40 dark cells (Figs. 4b, 5b). Stichocyte with large centrally located nucleus, dense granules and relatively large vacuoles (Figs. 4b, d, 5a, b). Nerve ring 83±4µm (1.5% of total body length) from anterior extremity (Figs. 4a, 5a). Maximum width of bacillary band 24±7 µm; single row of bacillary cells at anterior body region and 3-5 rows at middle and posterior body regions (Figs. 4c, 6b). Pores of bacillary cells deeply inserted in cuticle (Fig. 6b). Intestine wide straight tube, followed by a narrow convoluted one close to distal region of uterus (Figs. 4d, 5c). Two large wing-like cells present at junction between glandular oesophagus and intestine (Figs. 4d, 5c).

Ovary germinal zone with small oogonia lying along one side of ovary (Fig. 4e). Growth zone filled with packed rectangular, or cuboidal oocytes (Fig. 4e). Immature eggs 35 ±7 µm long and 21±3 µm breadth, with thin eggshells and incompletely formed polar plugs. Mature eggs 52±10 µm long and 25±2 µm breadth, arranged in single or sometimes two rows (Fig. 5d). Eggshell of cleaved mature egg differentiated into 3 distinct layers: outer thick, middle hyaline and inner thin (Figs. 4f, 5e, f). Mature egg possessing 2 distinct pale nuclei, 2 polar plugs and filled with dense granules (Figs. 4f, 5e) Some mature eggs in terminal portion of uterus have 2 unequal blastomeres (Figs. 4f, 5e). Vagina long, wide muscular tube and vulva with slightly elevated anterior lip (Figs. 4d, 5c, d,6c). Vulva slit-like opening, lying ventrally 1505±490 µm from anterior extremity and 35±10 µm posterior to oesophagus (Figs. 4d, 5c, 6c). With SEM, anterior lip covered with corrugated cuticular folds, posterior lip smooth (Fig. 5c). Caudal end rounded, with relatively long groove, surrounded by two smooth surfaced lobes, one larger than other (Fig. 6d). Posterior body region covered by cuticular transverse striations (Fig. 6d).

## Discussion

A redescription has been done for the nematode *C. fritschi* infecting the intestine of the catfish *B. bajad* and *B. docmak* (Bagridae) from the Damietta branch of the River Nile. This is the first time to describe this nematode using Scanning Electron Microscopy (SEM) and provide more additional morphological data of the cephalic and caudal regions, digestive system and reproductive system including invaginated and evaginated cirrus and vulva. The present morphometrical data of *C. fritschi* resemble, in general, those reported by Moravec (2001) (see Table 1) with minor variations which appear to be intraspecific. The number of stichocytes, in the present study, ranges between 35- 44 while that of Moravec (2001) is 27-35. This difference could be attributed to the number and size of specimens examined. The vulvar lips, in the present study, are slightly elevated particularly the anterior lip. In the present specimens, some mature eggs are cleaved and possess eggshells differentiated into 3 layers; 2 layers by Moravec (2001).

**Table 1:** Comparison between measurements (in µm) of the present study and those of Moravec (2001) of the nematode, *Capillostrongyloides fritschi*.

Female	Present study	Moravec, 2001	Male	Present study	Moravec, 2001
Total length	5440±1770	6070-9320	Total length	3564±608	3860-4420
Anterior width	38±13	−	Anterior width	21 ±12	–
Maximum width	55±23	54–81	Maximum width	37±20	41-54
Muscular oesophagus	185±82	189-330	Width of bacillary band	12±5	9
Glandular oesophagus	1962±1320	2240-3020	Muscular oesophagus	202±16	201-240
Bacillary band width	24±7	21-30	Glandular oesophagus	1608±129	1800-1840
Distance of nerve ring from anterior extremity	83±4	72-90	Nerve ring position	55±10	51-84
Distance of vulva from end of oesophagus	57±27	3-60	Spicule length	195±45	267-270
Ova length width	52±10 25±2	51-66 27-36	Spicule width	4.3±1.5	-
			Cirrus invaginated Cirrus evaginated	477±40 223±31	-
			Cirrus long tube Bulb Short tube Funnel-shaped region	75±15 40±6 21±6 23	- - -

Most species of *Capillostrongyloides* exhibit a rather uniform morphology and are mainly differentiated by the body length, the length of spicules, the size and shape of the evaginated spicular sheath (cirrus), the shape and length of the caudal lobes and membranous bursa, and presence/absence of elevated vulvar lips. Few of the previous studies on *Capillostrongyloides* species (Moravec, 2001; González-Solís et al., 2014) have described the male copulatory organ (cirrus) in the evaginated state. In the present study, the cirrus could be detected in successive stages (patterns) of evagination, and the structure of a completely everted cirrus is described in detail using phase contrast and incompletely everted by SEM. The completely evaginated cirrus consists of three main regions: the proximal long tube, the middle spherical bulb, and the distal funnel-shaped structure. Part of the terminal portion of the ejaculatory duct and spicule extend inside the proximal region where the ejaculatory duct dilates into a spacious cavity packed with mature spermatozoa in the bulb region. In the original description of *C. fritschi* made by Travassos (1914) and the description made by Moravec (2001), evaginated cirrus was not detected or described. In the present study, the evaginated cirrus of *C. fritschi* is unique among other described *Capillostrongyloides* species or indeed among other species belonging to the family Capillaridae. Among 10 previously described species of the genus *Capillostrongyloides,* only two species were reported to show evaginated cirrus, *C*. *ancistri* (see Moravec, 1987), and *C*. *morae* (see González-Solís et al., 2014). In both species, the evaginated cirrus is a cylindrical tube without a bulb or funnel-shaped structure that is present in the cirrus of *C. fritschi*. Variations have been reported in the structure of the cirrus in other members of the family Capillaridae. In *Paracapillaroides acanthocotylus* Rossin & Timi, 2009**,** evaginated cirrus consists of a short, cup-shaped, spinose, proximal part and a long, narrower, tubular, non-spiny distal part, indicating the absence of the spherical bulb and the funnel-shaped distal region. The evaginated cirrus is an ampulla-shaped structure in *Amphibiocapillaria combesi* Chabaud & Knoepffleri, 1985, a very long cylindrical tube with protruding spicule in *Capillaria* (*Capilloroides*) *catenate* Van Cleave & Mueller, 1932, *Amphibiocapillaria tritoniscnstati* (Diesing, 1861) Moravec, 1982, and *A. tritonispunctati* (Diesing, 1851) Moravec, 1982, a very long tubular structure without spicule in *Paracapillaria (Ophidiocapillaria) cesarpintoi* (Freitas & Lent, 1931) Moravec, 1986, a very long tube with a globular expansion at its proximal end in *Lobocapillaria austropacifica* (see Moravec & Beveridge, 2017) or a bell-shaped and relatively short cylindrical tube with the spicule projecting from its opening in *Paratrichosoma recurvum* (Solger, 1877) Ashford & Muller, 1978. As in the present study, most of the previously described evaginated cirri are covered with transverse cuticular folds (e.g., *Pseudocapillaria* (*Pseudocapillaria*) *carangi* (Parukhin, 1971) Moravec, 1982.

The mechanism of copulation has not been observed in living *C. fritschi* or in other capillarids. Most male nematodes with spicules at their posterior region are used to spread and open the vulva and vagina during copulation, with the aid of the other accessory reproductive structures (Wharton, 1986). In male nematodes which lack spicules as in the roundworm *Myolaimus,* sperm transfer involves at least six different secretions of the male gonoduct that are pumped into a sack-like cuticular protrusion of the female's vulva to form a spermatophore-like capsule (Fürst von Lieven et al., 2005). In the present study, most of the whole length of the spicule remains inside the body with only a short part of its distal region protruding with the evaginated cirrus. The everted cirrus of *C. fritschi*, is supported by transverse cuticular folds at the proximal and middle regions as well as fine and thick transverse and longitudinal muscle fibers along its length. It seems possible that these cuticular folds serve to keep the rigidity of the everted cirrus and prevent it from being collapsed during copulation. Moreover, contraction of the transverse muscles and relaxation of the longitudinal muscles may serve to elongate the cirrus and enable the funnel-shaped terminal end to enclose the lips of the vulva and make a firm attachment. Meanwhile, contraction of the transverse muscles around the bulb and relaxation of the sphincter muscles of the tube connecting the bulb with the funnel can create great pressure on the bulb cavity and leads to the movement of the mature spermatozoa into the funnel and subsequently to the vulvar opening. Contraction of the longitudinal muscles and relaxation of the transverse muscles can lead to the shortening of the cirrus and withdrawal of the funnel. The posterior body region of *C. fritschi* is supported by accessory genital organs like the membranous bursa, the ventrolateral lobes and posteriorly directed papillae. The real functions of these organs are not known but seem likely to secure a firm attachment of the male posterior region to the female body, thus facilitating the emergence of the cirrus during copulation. Also, contraction of the transverse circumferential muscles encircling the posterior body region may assist in the projection of the cirrus from the cloacal opening as the first step in copulation. In all these steps, a small part of the spicule was seen in the proximal region of the cirrus indicating that the spicule moves outside the cloacal opening and contributes to the emergence mechanism of the cirrus but has no role in widening the vulva during copulation as suggested for spicules in many other nematodes (Fürst von Lieven et al., 2005). For a concise understanding of the copulation process, living males and females might be examined while copulating using a stereomicroscope and SEM. Moreover, the musculature of the copulatory organs in both males and females should be studied in detail with the fluorescent and confocal laser scanning microscope to show the different types of muscles involved in the copulation process.

In the original description of *C. fritschi* made by Travassos (1914) and the description made by Moravec (2001), the bacillary bands are covered by tiny papillae-like formations and the mouth has no stylet while in the present study, the stylet is present, but papillae-like formations have not been detected either with the light microscope or SEM. The stylet was not described in all adult species of the genus *Capillostrongyloides* (see Santos et al., 2008 and Cantatore et al., 2009) except *C. ancistri* (see Moravec, 1987) and *C. morae* (see González-Solís et al., 2014). However, the first-stage larvae of *Trichuris trichiura* Linnaeus, 1771, *Trichinella spiralis* Owen, 1835 and *Trichosomoides crassicauda* (Bellingham) were shown to have distinct stylets, a feature which led Anderson (1992) to believe that the presence of a stylet in the first-stage larva is characteristic of all trichinelloids. In capillariids, Wright (1974) observed the stylet in the buccal cavity of adult *Calodium hepaticum* Bancroft, 1893 and suggested that the stylet can probably remain without projection from the mouth (Wright, 1961). However, a functional stylet was observed by Baruš et al. (1981) in adult *Capillaria anatis* Schrank, 1790 and by Moravec & Justine (2014) in *Capillaria plectropomi*. The finding of functional stylets in the previously mentioned capillariids suggests its presence in all adult capillariids (Moravec & Justine, 2014). The stylets in adult nematodes may serve in the withdrawal of food and could share in the metabolic process (Moravec, 2001).

The cephalic region of *C. fritschi* closely resembles that of *C. morae* described by González-Solís et al. (2014), *C. anatis* Schrank, 1790 by Baruš et al. (1981), *Paracapillaria philippinensis* Chitwood, Velasquez & Salazar, 1968 by Moravec (2001), *C. plectropomi* by Moravec & Justine (2014), *Capillaria appendigera* n. sp. (see Moravec & Barton, 2018), *Paracapillaria gastrica* n. sp. (see Moravec & Justine 2020), except for the amphids and oral lips which are not clearly visible and the cephalic papillae which are lesser in number and buried in the cuticular surface. In these capillariids, 12 cephalic papillae (sessile or buried) were observed (distributed in two circlets) on the cephalic plate around the oral aperture. Regarding their position in the cephalic region, these papillae may act as chemoreceptors.

## Data Availability

All data used in this study are available as soon as requested.
